# The durability of mechanical properties of flexible waterproofing membranes used on ventilated terraces under long term exposure to high temperatures

**DOI:** 10.1038/s41598-025-05007-y

**Published:** 2025-06-03

**Authors:** Barbara Francke, Ewelina Kozikowska, Ewa Sudoł, Marta Zawadzka, Vadim Griniov, Agnieszka Starzyk

**Affiliations:** 1 Institute of Civil Engineering, Warsaw University of Life Sciences-SGGW, Nowoursynowska 159, 02-787 Warsaw, Poland; 2 Building Research Institute, Filtrowa 1, 00-611 Warsaw, Poland

**Keywords:** Ventilated terraces, Waterproofing membranes, Reinforced bitumen sheets, EPDM membrane, Mechanical strength, Durability under thermal exposure, Engineering, Materials science

## Abstract

The paper analyses the influence of high temperatures (70 °C for three months) on the durability of the mechanical properties of waterproofing membranes used on ventilated terraces. The test kits consisted of a concrete substrate, waterproofing membrane (reinforced bitumen sheet or EPDM membrane), and the top layer spot-supported by plastic pedestals. The stress transferred by the pedestals to the substrate was determined through calculations. The changes after ageing were evaluated in the areas between the pedestals and in their locations for their ability to maintain the mechanical load-transferring function, including but not limited to resistance to continuous or instant spot loads. The action mechanism of the ageing processes was investigated by analysing the microstructure of the cross-sections of the tested samples using SEM. After three months of exposure to + 70 °C, the resistance to spot loads of a reinforced bitumen sheet was higher than that of a flexible EPDM sheet. Simultaneously, the tensile values are more favourable for the first sheet type. The impact resistance of the reinforced bitumen sheet increased by 36%, whereas that of the EPDM membrane decreased by 25%. The static puncture resistance of the reinforced bitumen sheet before and after ageing remained at the same level; for the EPDM membrane, the value dropped by 20%. In both cases, microscopic images of the cross-sections of the tested items revealed structural changes in the form of microcracks.

## Introduction

**Fig. 1:**
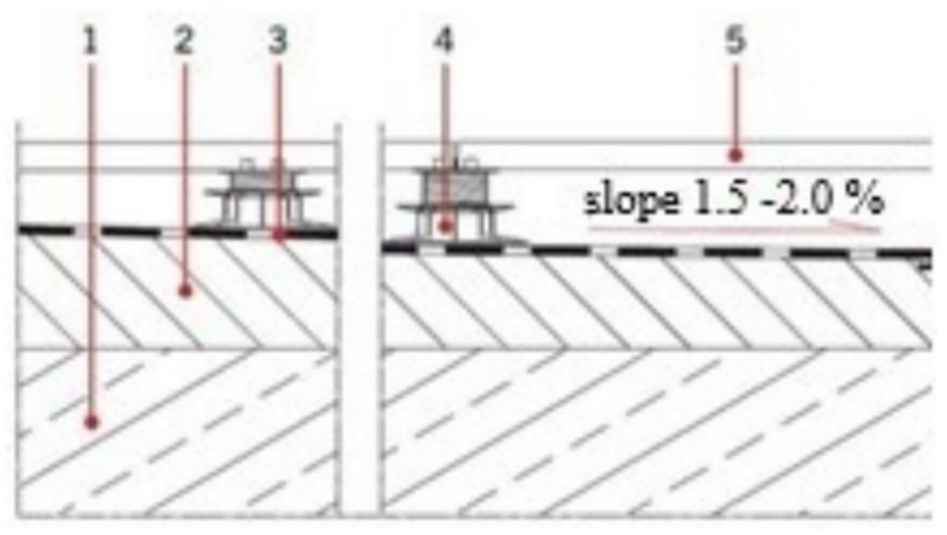
Ventilated terrace decking: cross-section. Marking: 1—load-bearing layer, i.e. reinforced concrete construction slab, 2—slope formation layer, 3—waterproofing layers from a flexible product, 4—adjustable pedestals, 5—top layer.

Most processes that destroy building materials occur in the presence of water or moisture; therefore, structures must be protected from the ingress of unwanted rainwater or water accumulated in the ground^1,2^. Such protection makes it possible to guarantee the comfort of using indoor spaces. In the case of buildings, it indirectly affects the health and lives of their occupants. Each building should be protected against the ingress of unwanted rainwater or water accumulated in the ground or on the surface of terraces and balconies, as well as water splashed on the floor of “wet” rooms and delivered there from the plumbing system^2^. To meet the requirement and guarantee the longest possible maintenance-free service, the arrangement of layers in each structure should be designed and executed to fulfil its intended function failure-free in the designed service life. This study is devoted to assessing the durability of one of the above-mentioned waterproofing application areas, that is, waterproofing layers of terraces and balconies, assuming that the durability of this part of the covering determines the proper protection of the structure against water and moisture. Because a terrace often constitutes decking for the premises below, its structure and workmanship shall provide durable protection against atmospheric precipitation and ensure adequate thermal comfort indoors. Terrace decking can be constructed as a traditional system, that is, with a thermal insulating layer under the waterproofing membrane^2^, or as a reverse system, where the thermal insulation is laid on the waterproofing membrane^2,3^. This study focuses on a standard solution in which waterproofing layers are glued to the substrate and the top layers rest on their surface. The top layers can be glued to the adequately protected waterproofing layers or rest on plastic pedestals^4^, typically made of polyethene or polypropylene, forming a ventilated air void between the top and waterproofing layers. Pedestals come in various types, from simple to adjustable, enabling smooth level adjustment, levelling the usable layer, or elevating the flooring by several centimetres versus the waterproofing level. The surfaces in such solutions are composed of small items capable of transferring the loads they are exposed to during service without any damage. To this end, ceramic tiles sized 60 cm × 60 cm and at least 2 cm thick, or concrete pavement slabs of various sizes, are typically used. A sample solution is illustrated in Fig. 1.

In the solutions above, the waterproofing layers are exposed to numerous factors, such as climatic factors, for example, varying negative and positive temperatures^5–9^ in the presence of spot static loads and operating dynamic loads transferred by the pedestals from the surface onto the waterproofing layers. There are also influences related to snow removal from terraces and balconies and other maintenance and repair works that often cause mechanical damage by static and dynamic loads^10^. These additional impacts are difficult to predict and, therefore, cannot be classified as cyclic loads, which compromises the analysis of this phenomenon as a function of durability.

Waterproofing layers of terraces and balconies can be made of various materials, including waterproofing membranes made of cement-based materials containing various modifiers, polymer coats, and flexible waterproofing sheets, that is, reinforced bitumen sheets^11^ or plastic and rubber membranes, laid over concrete or cement mortar substrate. The studies presented in this manuscript apply to the latter two, that is, reinforced bitumen sheets and EPDM membrane. Technical publications present numerous results for both product groups concerning various durability aspects of bituminous waterproofing membranes^11^ and rubber membranes^13^.

Bituminous materials are among the most popular materials used in civil engineering. It is viscoelastic material obtained through vacuum distillation of crude oil^14,15^. Its impermeability to water makes it an excellent waterproof material, particularly in buildings^16^. However, water infiltration may accelerate bitumen ageing and reduce binder adhesion between the bituminous coating and other materials, especially at low temperatures in winter^17^. A decrease in interfacial adhesion can directly affect the damage and durability of the bituminous coating^18^, accelerating the cracking of bitumen and leading to a loss of waterproofing function^19–22^. Bitumen is the primary waterproofing agent in reinforced bitumen sheets. They are ready-made flexible sheets, including carriers/reinforcements, bitumen coatings, surface textures, and/or backing. The top surface of the bitumen coating is covered by a finishing layer which protects the sheet against weathering, for example, fine or coarse mineral granulates. The underside is protected by an anti-sticking substance for transport and/or storage purposes. Reinforced bitumen sheets are supplied in flexible sheets and are ready to use^11^. Studies on bituminous waterproofing membranes concerning, for example, their dimensional stability^23–25^, the adhesion of self-protection mineral granules^25^, the effects of soil radon and bacteria on the mechanical properties of the membranes^26^, or the adhesion properties and debonding failure modes of bituminous waterproof materials for cement substrates^27^ are worth mentioning.

Ethylene propylene diene monomer (EPDM) rubber is a polymer composed of ethylene, propylene, and a third monomer, and is considered one of the most rapidly advancing synthetic rubbers^28,29^. Renowned for its outstanding chemical stability, water resistance, low-temperature tolerance, and electrical insulation properties, EPDM rubber is broadly used in various industries for automotive parts, wires, cables, building and structure waterproofing^30,31^, and sealing^32–34^. Rubber materials have also been extensively tested for artificial accelerated ageing. The Arrhenius equation helps establish a conversion relationship between accelerated ageing tests and actual service conditions^35^. Several researchers have utilised tensile properties, such as tensile strength and elongation at break, as ageing indices to predict the durability of EPDM materials^13,36,37^.

The research discussed in this study aimed to determine the following:

- whether the waterproofing layers typically made of flexible products such as reinforced bitumen sheet or EPDM membrane can transfer the loads transmitted by the pedestals on which the top layers of ventilated terraces are laid, with no damage once exposed to high temperatures,
- how the structure of the waterproofing products changes after the referenced exposure,
- whether the potential damage to the waterproofing layers caused by service loads does not compromise their primary function, that is the watertightness of the terrace decking.

According to the literature, the maximum usable temperature of terrace surfaces is + 70 °C. This temperature was used in the ageing tests performed in this study. The test temperature was set based on the literature data^5–9,38^ and the test results were used to develop European testing standards for evaluating the durability of waterproofing sheets after exposure to high temperatures (EN 1296^39^). Because the temperatures to which the terraces are exposed vary depending on the building location and climate variability, including the solar radiation energy changes throughout the year, as well as the colour of the top layer, the study authors decided to adopt the maximum value quoted in the referenced technical studies.

## Materials

Based on the initial elimination tests performed for different terrace systems, two waterproofing materials were selected for further ageing tests, which were common for the following material groups:

- EPDM membrane (terpolymer of ethylene, propylene and a diene with residual unsaturated portion of diene in the side chain membrane), 1.2 mm thick, 1.23 kg/m^2^ weight. After twelve weeks of thermal ageing at + 70 °C, the product flexibility did not change compared to the pre-exposure condition. In none of the cases was the membrane damaged at bending at − 40 °C,
- Reinforced bitumen sheet for waterproofing on a polyester non-woven reinforcement, 5.2 mm thick and 250 g/m^2^ weight, reinforced and stabilised with a glass mesh, with an SBS-modified bitumen mix coat on both sides. The top surface of the reinforced bitumen sheet is coarse and granular. The underside was covered with a red acrylic coat. After twelve weeks of thermal ageing at + 70 °C, the product flexibility did not change compared to the pre-exposure condition. In none of the cases was the reinforced bitumen sheet damaged by bending at − 15 °C.

**Fig. 2:**
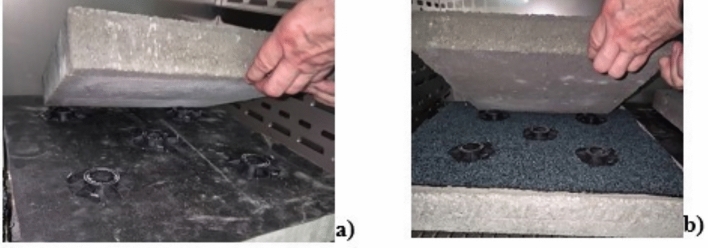
Method of laying the top layer on the waterproofing membrane made of a) EPDM, b) reinforced bitumen sheet.

To reflect the service conditions of a ventilated terrace surface under mini-scale thermal exposure, that is, in laboratory conditions, the test kits composed of the following were used (in their laying sequence):

- concrete substrate,
- waterproofing layer made of EPDM membrane or reinforced bitumen sheet,
- top layer laid loosely on five pedestals arranged in the specimen’s four corners and in the middle—at the intersection of the slab diagonals (Fig. 2).

**Fig. 3:**
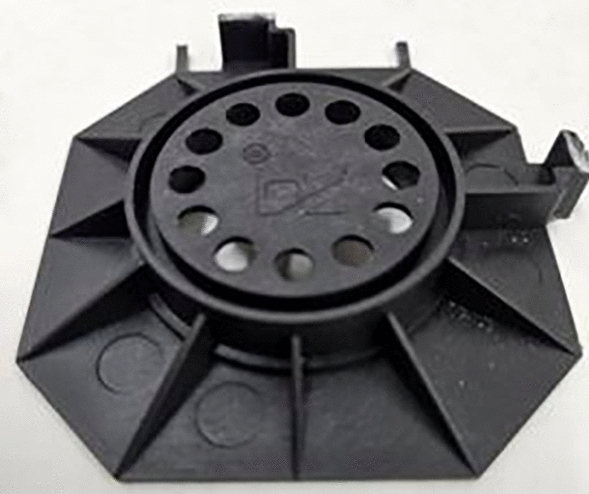
View of a PP pedestal.

The following complementary products were used to prepare the test kits:

- substrates—concrete slabs, C20/25 grade, size: 0.07 m × 0.5 m × 0.5 m, destructive load rating 140(14), characteristic non-destructive load 14.5 kN, minimum destructive load 11.6 kN, single slab weight ca. 39 kg,
- stiff polypropylene (PP) pedestals for laying the top layer, each weighing 41 g and 10 mm high, with a square base with bevelled corners, surface dimensions ca. [(70 mm × 70 mm)−(15 mm × 15 mm)] and a 40 mm hole in the centre (Fig. 3).
- top layer made of C20/25-grade concrete slabs, size: 0.06 m × 0.35 m × 0.35 m, destructive load rating 70(7), characteristic non-destructive load 11.0 kN, minimum destructive load 8.8 kN, single slab weight ca. 14 kg.

## Test methods

### Ageing exposure

**Fig. 4:**
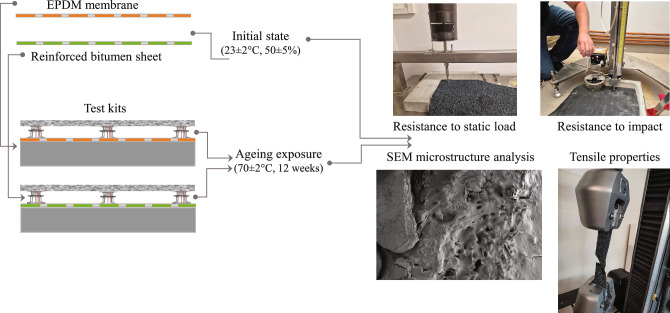
Experimental flowchart.

Ageing tests were aimed at determining the changes occurring in waterproofing layers ventilated as a result of exposure to high temperatures. Simultaneously, the layer was exposed to loads transferred onto its surface by pedestals. To that end, the test kits were exposed to (70 ± 2) °C for 12 weeks in a Pol-Eko SLW400 climatic chamber with forced airflow. After ageing, macroscopic evaluation was performed with the naked eye from a 50 cm distance for potential surface damage and defects. The resistance to static load, impact resistance, and microstructure were then analysed (Fig. 4), which are vital for layer durability. The twelve weeks were selected based on previous research experience used in developing European standards for evaluating the durability of flexible sheets once exposed to high temperatures, according to the methodology described in EN 1296^39^.

### Microstructure SEM analysis

The microstructure of the EPDM membrane and reinforced bitumen sheet was investigated using a Sigma 500 VP (Carl Zeiss Microscopy GmbH, Köln, Germany) Scanning Electron Microscope (SEM) with cold field emission. The tests were performed at 5 keV accelerating voltage, using an SE detector, on samples sprayed with gold coat.

The microstructures of the EPDM membrane and reinforced bitumen sheet cross-section surface were observed before the ageing exposure at × 100 and × 200 magnification, and the EPDM membrane was analysed at × 1000 and × 5000 magnification.

### Tensile properties

The tensile properties of the EPDM membrane were tested according to EN 12311-1:1999^40^, and those of the reinforced bitumen sheet were tested according to EN 12311-2:2013^41^. The samples in both test cases were 200 ± 15 mm long and 50 ± 0.5 mm wide. The tests were carried out in a computer-controlled INSTRON 5966 strength testing machine that featured a 10 kN capacity head and an optical extensometer for elongation measurements. The feed rate was 100 ± 10 mm/min. The tests were performed in standardised laboratory conditions on the samples before ageing exposure and collected from the test pieces after ageing exposure, from the areas between the pedestals. Five samples were used for each series. The tensile strength was determined, expressed in MPa, as the ratio of the maximum force value to the sample’s cross-section. Elongation was simultaneously monitored on a 100 ± 15 mm measurement section. The elongation value recorded at the maximum force was referred to as the measurement section’s initial value, obtaining the relative elongation (expressed in %) at the maximum force.

### Resistance to static load and impact resistance

Resistance to static load and impact resistance were tested at room temperature (23 ± 2)°C on samples before and after ageing exposure. The samples after ageing exposure were obtained after dismantling the test kits, from the areas between the pedestals.

Resistance to static load was tested according to EN 12730^42^, using a hard support i.e. concrete. The test involved applying spot loads of 5, 10, 15, and 20 kg (successively) to the waterproofing product surface; the extra load for the EPDM was 25 kg. The loading was executed with the weight of a steel ball at the bottom (10 mm diameter and 50 HRC hardness). The load was maintained for 24 h. Successive loads were applied to different sample areas five times for each weight. The maximum load weight in kg whose impact did not cause a puncture in at least three of five tests was considered a positive result. The puncture was evaluated using a vacuum device. The sample surface was covered with water mixed with a surfactant within less than five minutes of removing the load. The tightness (lack of air bubbles) was checked at a 15 kPa differential pressure.

The impact resistance was tested according to EN 12691^43^ using a hard substrate, i.e. concrete. The test involved hitting the upper surface of the waterproofing product with a free-falling weight (500 ± 5 g) with a steel ball at the bottom (12.7 ± 0.1 mm and 50 HRC hardness). The test was conducted on a test stand, allowing free fall of the weight perpendicularly to the sample plane. The sample was protected against displacement using a ring (2 kg weight and 100 mm inner diameter). Successive impacts were executed in different areas of the sample five times for each weight falling height. The falling height was the distance between the bottom of the ball and the surface of the sample. The falling height (in mm) at which no puncture was observed in at least four out of five tests was the test result. The puncture was evaluated using a vacuum device, similar to the method used in the resistance to static load test.

## Results and discussion

**Fig. 5:**
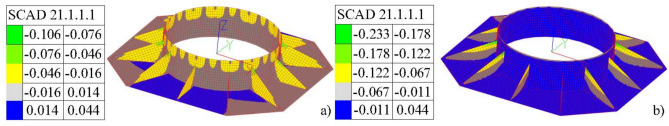
Stress distribution within the middle pedestal: (**a**) stress exerted by standard forces along the X axis, **(b**) stress exerted by standard forces along the Y axis.

Testing the resistance of ventilated terrace (i.e. with top layers laid on pedestals) waterproofing membranes to long-term exposure to high temperatures involved analysing the behaviour of waterproofing materials in the pedestal locations. The option adopted for the analyses was the same as in the test kits, with the top layer of a concrete paving slab (0.06 m × 0.35 m × 0.35 m, 14 kg weight) resting on five plastic pedestals. One pedestal was placed at the intersection of the axes in the centre of the top layer, and the other four were arranged in the corners of the paving slab. The contact area between each pedestal and waterproofing membrane resembled a 1.5 cm wide ring. Based on computer simulations performed in SCAD^44^, it was determined that the force transferred by the middle pedestal was the highest and amounted to 57 N at a mean pressure value of 41.2 kPa exerted on the uppermost surface of the 1385 mm^2^ substrate area. Each edge pedestal transferred much lower forces of approximately 20 N. The circumferences with stress isopoles on the surface of the axially loaded middle pedestal are shown in Fig. 5. The pedestal was assumed to rest on a substrate with a modulus of elasticity characteristic of the waterproofing material. In the computer simulation, the pedestal model was divided into 7998 tiles.

**Fig. 6:**
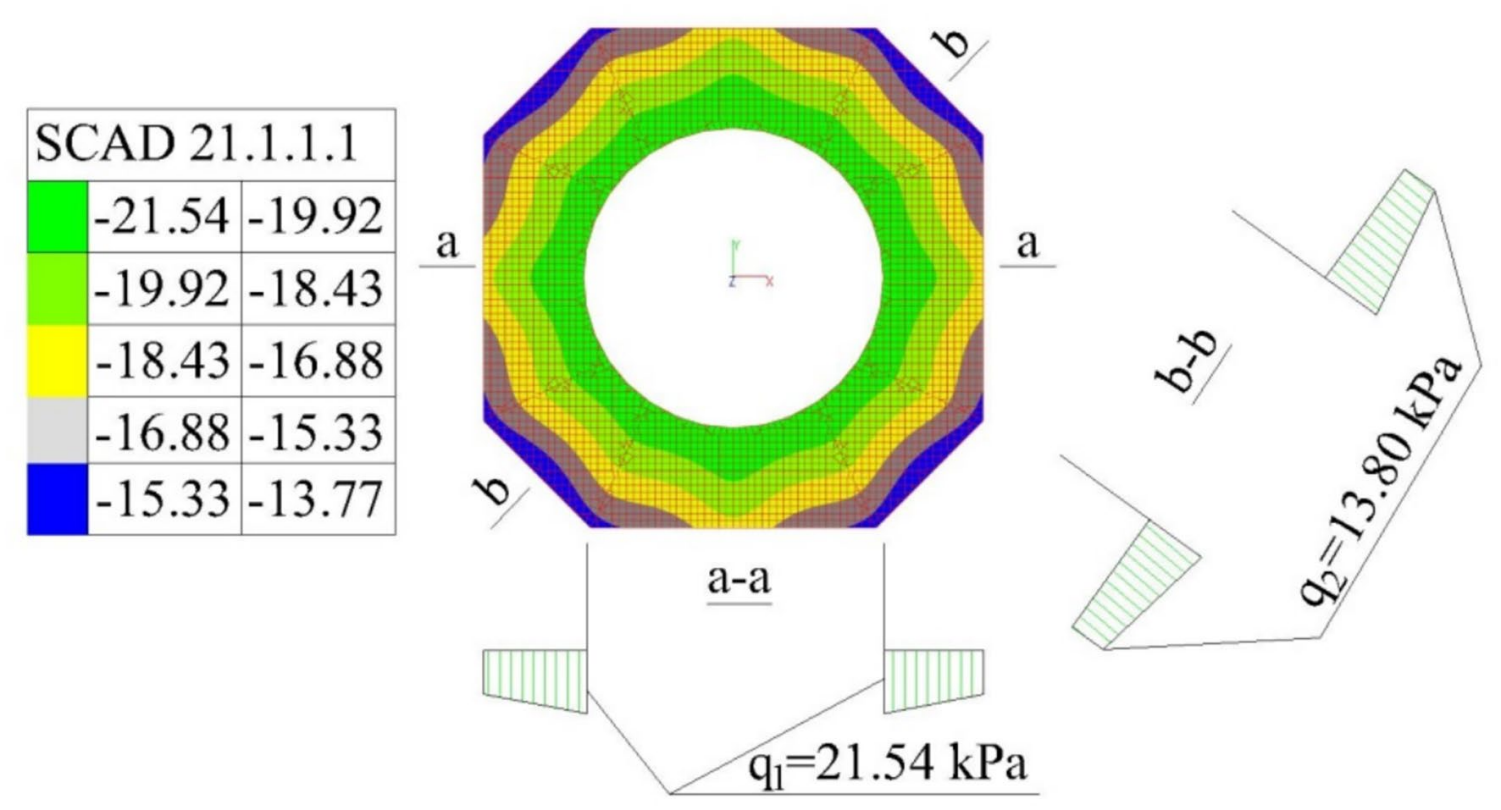
Stress distribution after substrate reaction under the middle pedestal, including stress values in the A-A and B-B section.

The stress exerted on the substrate in response to the middle pedestal pressure is shown in Fig. 6. The stress under the middle pedestal was identified to range between 13.8 kPa and 24.4 kPa.

**Fig. 7:**
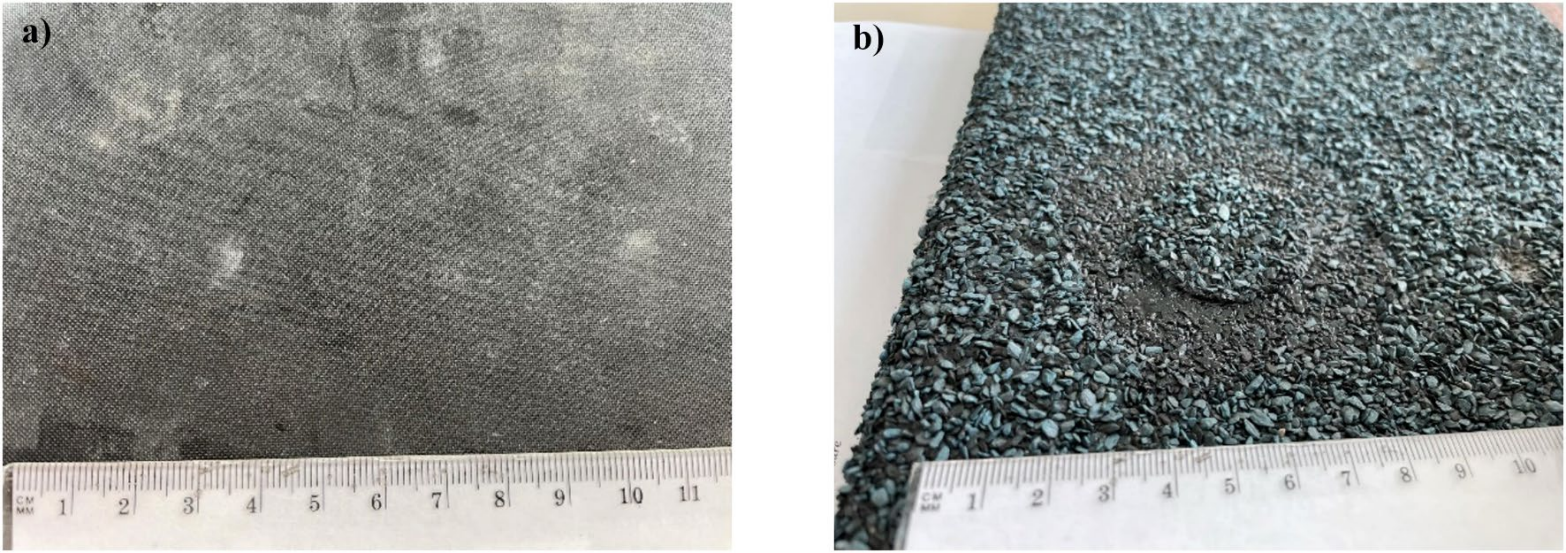
Appearance after ageing exposure in pedestal locations: (**a**) EPDM membrane, (**b**) reinforced bitumen sheet.

After 12 weeks of load exposure and simultaneous ageing exposure at 70 °C, no visible changes were observed in the macroscopic evaluation of the EPDM waterproofing membrane surface damage (Fig. 7a). The observed surface damage of the reinforced bitumen sheet involved granular coat indentation in the pedestal areas and simultaneous uncovering of the bitumen sheet surface mix layer (Fig. 7b).

The damage described above does not compromise the waterproofing membrane’s integrity, which means that watertightness is maintained. To describe the mechanism of the phenomenon, a comparative analysis of the referenced damage was performed using SEM images. The process of changing the asphalt-polymer mix structure has been analysed in the technical literature primarily for road asphalts after heating the test samples to at least 180 °C^45–50^, which significantly changes the phase structure of the tested systems. Microstructure analyses of industrial asphalts used for producing bitumen sheet coating mixes after long-lasting exposure to + 70 °C, presented in this article, have not been the subject of deliberations.

**Fig. 8:**
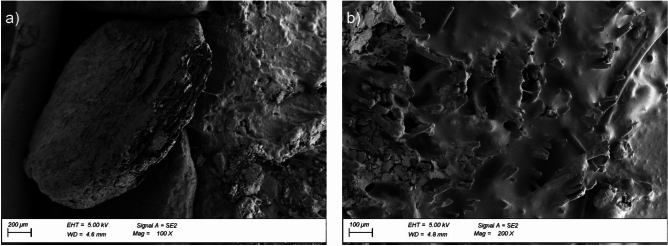
Microstructure of the reinforced bitumen sheet cross-section before ageing exposure: (**a**) granulate with a fragment of the coating asphalt-polymer mix, magnification × 100, (**b**) fragment of the coating asphalt-polymer mix, magnification: × 200.

Morphology analysis involved a qualitative evaluation of the cross-sectional surface of the reinforced bitumen sheet, verification of the changes occurring at the border between the mineral granulate and the asphalt-polymer coating mix, and direct evaluation of the asphalt-polymer mix damage. The cross-sectional surface of the reinforced bitumen sheet was compared before ageing exposure, after thermal ageing (samples from the areas between the pedestals), and after thermal ageing combined with static loading (samples from the areas under the pedestals). The microstructure of the cross-sectional surface of the reinforced bitumen sheet samples before exposure revealed poor adhesion of the mineral granulate to the asphalt layers (Fig. 8a). The tested asphalt-polymer system was a two-phase system, where the asphalt was the continuous phase, similar to other cases analysed in the literature^51^. Uniformly arranged small round particles of styrene–butadiene–styrene copolymer were also observed. Observations of the asphalt-polymer coating mix before exposure revealed uniform coating of the reinforcement by the asphalt mix. The images show fibres originating from a polyester non-woven reinforcement. The images of the pre-exposure samples did not show any cracks in the asphalt mix (Fig. 8b).

**Fig. 9:**
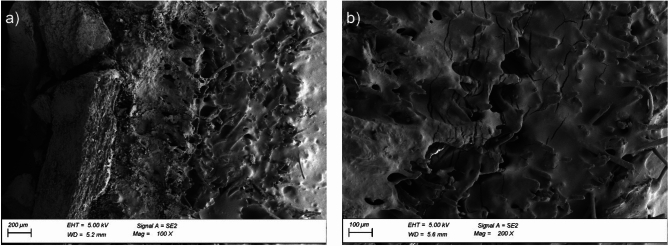
Microstructure of the reinforced bitumen sheet cross-section after ageing exposure: (**a**) granulate with a fragment of the coating asphalt-polymer mix, magnification × 100, (**b**) fragment of the coating asphalt-polymer mix, magnification: × 200.

**Fig. 10:**
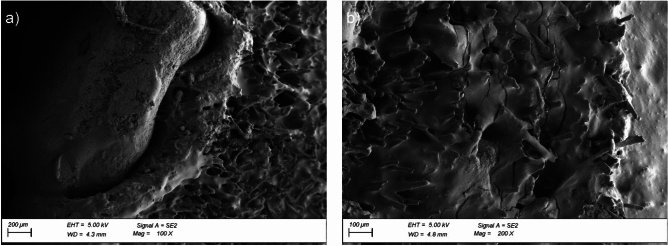
Microstructure of the reinforced bitumen sheet cross-section after ageing exposure in the pedestal area: (**a**) granulate with the coating asphalt-polymer mix, magnification × 100, (**b**) fragment of the coating asphalt-polymer mix, magnification: × 200.

Exposing the reinforced bitumen sheet to thermal ageing significantly changes the cross-sectional surface morphology. The microscopic images revealed a change in the adhesion nature of the mineral granules (aggregate); the granules were integrated with the asphalt mix (Fig. 9a). Numerous cracks were observed in the asphalt mix after thermal exposure (Fig. 9b). Although the technical literature mentions the potential self-healing of cracks in the asphalt mix structure, the process progresses more slowly in asphalt-polymer mixes than in pure asphalt mastic^52^. Uniformly distributed small, round particles of styrene–butadiene–styrene copolymer were still visible. Exposing the reinforced bitumen sheet to static load led to further integration of the granules with the asphalt-polymer mix (Fig. 10a) and an increasing number of cracks in the asphalt-polymer mix (Fig. 10b).

**Fig. 11:**
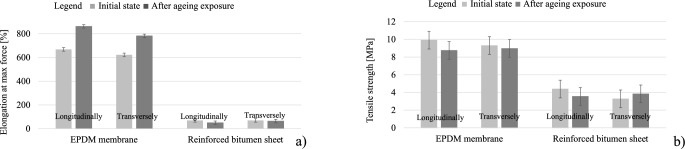
Tensile properties (**a**) elongation at maximum force, (**b**) tensile strength.

Homogenisation within the structure of the modified asphalt coating mix was apparent, and it progressed deeper into the layer. The internal structures become tighter and hence stabilise the system, improving its hardness and susceptibility to cracks oriented parallel to the outer edge of the reinforced bitumen sheet. These cracks occur within the asphalt (cohesive failure). This suggests the initiation of the colloidal transition of asphalt, which is the coating mix ingredient. The transition involves the successive transformation of asphalt oils into asphalt resins, which react with the resins used for asphalt modification in reinforced bitumen sheet production. The increase in coat hardness can also suggest the beginning of asphalt resin transformation into asphaltenes. However, the two processes are difficult to distinguish when asphalt modifiers are used. Long-term exposure to high temperatures deteriorated the flexibility of the bitumen sheet reinforcement made of polyester non-woven, which was demonstrated by a 28% decrease in the reinforced bitumen sheet elongation at break. For the reinforced bitumen sheet before exposure, the elongation was 70%, regardless of the testing direction. After ageing exposure, it was 50% longitudinally and 64% transversely (Fig. 11a). At longitudinal elongation, the maximum stress decreased from 4.4 to 3.6 MPa. For transverse elongation, the value increased slightly from 3.3 to 3.8 MPa (Fig. 11b). Values shown in the figures are the average of 5 measurements.

The morphology of the EPDM membrane was also analysed. The macroscopic evaluation did not reveal any changes (Fig. 7a). The analysis involved a qualitative assessment of the membrane’s cross-section before ageing exposure and verification of the changes in the membrane after thermal ageing (samples from the areas between the pedestals) and after thermal exposure combined with static loading (samples from the areas under the pedestals).

**Fig. 12:**
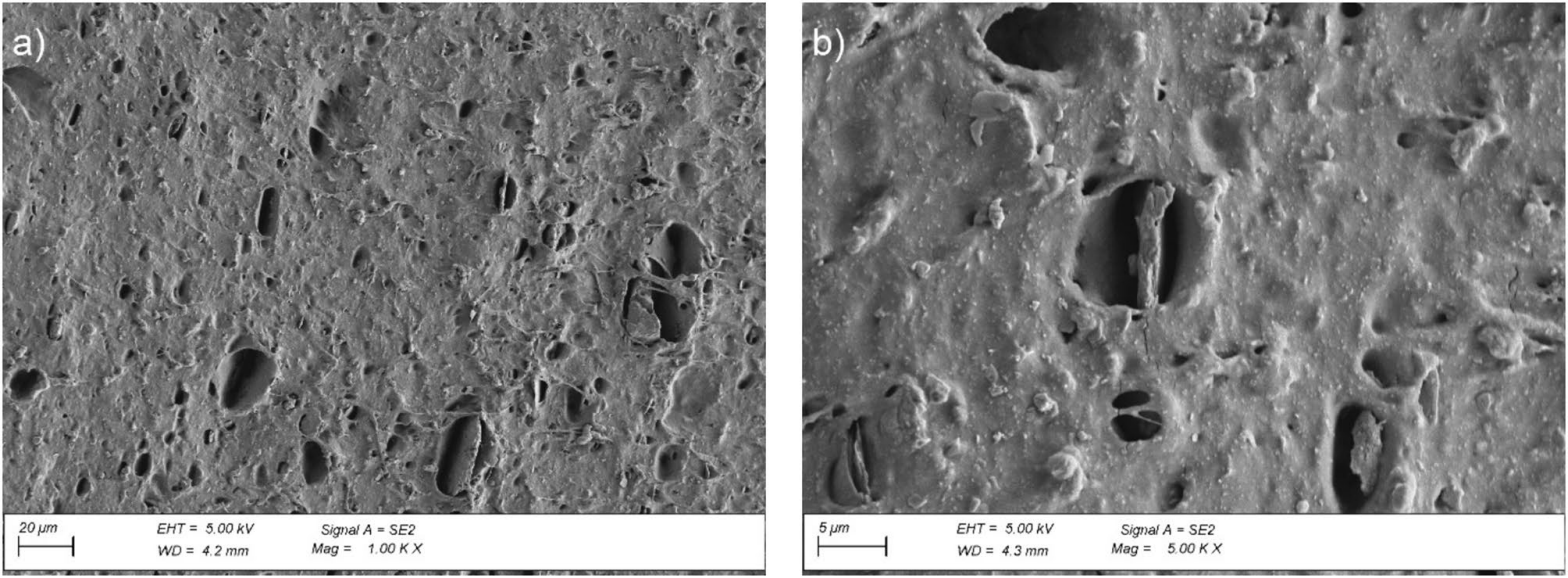
Microstructure of the EPDM membrane surface cross-section before ageing exposure; magnification: (**a**) × 1000, (**b**) × 5000.

**Fig. 13:**
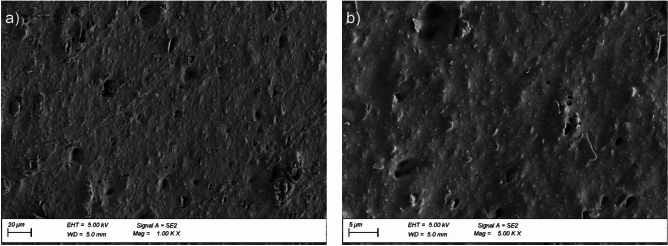
Microstructure of the EPDM membrane surface cross-section after ageing exposure; magnification: (**a**) × 1000, (**b**) × 5000.

An analysis of the surface microstructure of the EPDM membrane sample cross-section before exposure revealed a high number of pores over the entire analysed material surface (Fig. 12a, b). Additionally, the images show the particles of a mineral filler introduced into the EPDM membrane to reduce the amount of the flexible plastic component used. Thermal exposure to the EPDM membrane changes the cross-sectional surface nature and significantly reduces the number of pores in the material, as shown in Fig. 13a and b.

**Fig. 14:**
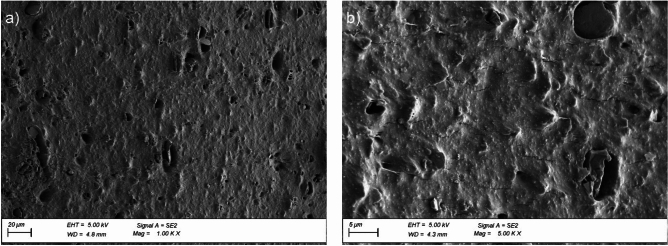
Surface microstructure of the EPDM membrane after ageing exposure in the pedestal location; magnification: (**a**) × 1000, (**b**) × 5000.

Exposing the EPDM membrane to static load at high temperature changes the pore content in the material and contributes to microcracks unnoticeable at × 1000 (Fig. 14a) magnification but apparent at × 5000 magnification (Fig. 14b). These observations suggest the first stage of degradation due to intensive exposure to high temperatures and higher availability of oxygen. The formation of microcracks facilitates further oxidation of the material interior, which deteriorates the longitudinal and transverse tensile strengths by 12% and 3%, respectively (Fig. 11a). Simultaneously, the plasticity, expressed as an increase in the relative elongation value at the maximum force, increased by 29% and 26%, respectively (Fig. 11b).

**Fig. 15:**
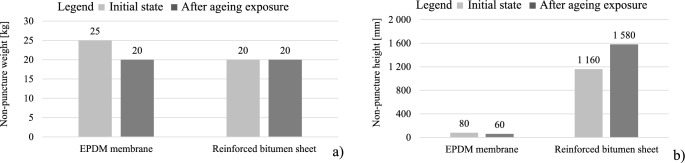
Puncture resistance of waterproofing products. (**a**) resistance to static load, (**b**) impact resistance.

In addition to the observations above, after long-term exposure to 70 °C, the waterproofing layer was evaluated for its resistance to static load (Fig. 15a) and impact resistance (Fig. 15b). The tests were performed in areas not subjected to the load transferred by the pedestals. The test results shown in the graphs determined by successive loads applied to different sample areas five times for each weight was reproducible. At all test points for the load value representing the test result, the membranes were tight. These results were confirmed during the evaluation of a vacuum device, finding no air bubbles at a pressure difference of 15 kPa. Therefore, the tests were not repeated and the variability of the results was not determined.

For the EPDM membrane, the results of the resistance to static load and impact resistance tests revealed a decrease in the tested parameter, corresponding to the direction of the tensile strength changes (Fig. 11b). The reduced number of pores observed in the SEM analysis deteriorated the product elasticity, which is the criterion for static and dynamic puncture resistance assessment. For the reinforced bitumen sheet, the microcracks formed within the structure of the modified asphalt coating mix at a simultaneous homogenisation of the coating mix internal structure, observed in the SEM image (Fig. 9b), did not deteriorate the tightness of the product. This was evidenced by the unchanged resistance to static load (Fig. 15a) and improved impact resistance (Fig. 15b).

## Conclusions

This paper presents the test results of selected mechanical properties of flexibles sheets of waterproofing materials used in ventilated terraces after long-term exposure to high temperatures. The temperature value in the tests was + 70 °C, and the exposure lasted three months according to the European standard recommendations for accelerated ageing tests in laboratory conditions. Simulation tests were conducted for systems representing the solutions used in building practice, which is, a concrete substrate was covered with a waterproofing layer on top, and the top layer was laid on five plastic pedestals. Reinforced bitumen sheets and EPDM foil were the waterproofing materials and 0.35 m × 0.35 m concrete tiles were the top layer.

Regarding the small population of the test objects, the results cannot be generalised but should be treated as significant manifestations requiring further studies. Considering the above, the following conclusions can be drawn regarding the durability of the mechanical properties of waterproofing flexible sheets used in ventilated terraces after three months of exposure to high temperatures:

- the durability of reinforced bitumen sheet mechanical properties is higher than that of flexible EPDM membrane after exposure to spot loads; the values obtained during stretching are also more favourable for the first material,
- the reinforced bitumen sheet impact resistance increased by 36% compared to the pre-exposure value (no puncture at the punch falling height of 1160 mm before and 1580 mm after ageing). For the EPDM membrane, the value dropped by 25% (80 mm before and 60 mm after ageing),
- static puncture resistance of the reinforced bitumen sheet before and after ageing remains at the same level (no damage for 20 kg load), whereas for the EPDM membrane, the value drops by 1/5 (25 kg before and 20 kg after ageing),
- as for the mechanical tensile properties, the plastic properties of the reinforced bitumen sheet deteriorated significantly, as the relative elongation at maximum stress decreased by 28.4% longitudinally and by 8.7% transversely. Simultaneously, the stress decreased by 19.1% longitudinally and increased by 17.1% transversely. The parameter suggests the temperature contribution to the changes occurring within the bitumen-sheet reinforcement. It is the primary contributor to the referenced functional parameter,
- as for the mechanical tensile properties, the EPDM membrane plasticity at stretching is much higher. This is evidenced by the increase in the relative elongation at maximum stress, that is, 29% longitudinally and 25.7% transversely. At the same time, the tensile stress decreases by 11.7% longitudinally and 3.4% transversely,
- thermal aging at + 70 °C had no effect on the change in elasticity of any of the products at negative temperatures, as confirmed by the flexibility test,
- the microscopic images revealed structural changes in both cases. For the reinforced bitumen sheet after thermal ageing, homogenisation was apparent within the modified asphalt coating mix and progressed deeper into the layer. The internal structures become tighter, and the system improves its stability, hardness, and susceptibility to cracking. The cracks were oriented parallel to the outer edge. They were more abundant at the pedestal locations. The new EPDM membrane has a highly porous internal structure, whereas the number of pores in the material decreases significantly after thermal ageing. This is accompanied by the formation of microcracks oriented perpendicularly to the outer edge.

## Data Availability

All data generated or analysed during this study are included in this published article.
